# Mild to moderate proteinuria is a heralding sign for acute kidney injury and mortality for intensive care unit patients

**DOI:** 10.3906/sag-1802-183

**Published:** 2019-04-18

**Authors:** Hasan H. YETER, Tolga YILDIRIM, Damla EYÜPOĞLU, Tural PAŞAYEV, Abdullah ASLAN, Sıla ÇETİK, Ömer AKÇAY, Arzu TOPELİ, Mustafa ARICI

**Affiliations:** 1 Department of Nephrology, Faculty of Medicine, Gazi University, Ankara Turkey; 2 Department of Nephrology, Faculty of Medicine, Hacettepe University, Ankara Turkey; 3 Department of Internal Medicine, Faculty of Medicine, Hacettepe University, Ankara Turkey; 4 Department of Internal Medicine, Zekai Tahiri Burak Hospital, Ankara Turkey; 5 Intensive Care Unit, Faculty of Medicine, Hacettepe University, Ankara Turkey

**Keywords:** Acute kidney injury, urine dipstick protein, proteinuria, 28-day mortality

## Abstract

**Background/aim:**

Lack of early predictors of acute kidney injury is currently delaying timely diagnosis.****This study was done to evaluate the relationship between mild to moderate proteinuria and incidence of acute kidney injury (AKI) and 28-day mortality in intensive care unit (ICU) patients.

**Material and methods:**

This observational, retrospective study was conducted in the internal medicine ICU. A total of 796 patients were screened and 525 patients were used for this analysis. Proteinuria was measured by urine dipstick test. AKI was defined according to the Kidney Disease: Improving Global Outcomes (KDIGO) guidelines.

**Results:**

Patients with dipstick urine protein positivity on admission had higher proportion of AKI and 28-day mortality compared to dipstick urine protein negative group [164 (59.6%) vs. 111 (44.4%) and 101 (36.7%) vs. 54 (21.6%), P = 0.01 and P < 0.01, respectively]. Urine dipstick protein positivity was also a significant predictor of 28-day mortality in patients with GFR > 60 mL/min (hazard ratio: 1.988, 95% confidence interval 1.380–2.862).

**Conclusion:**

Proteinuria before ICU admission is a risk factor for development of AKI within seven days of ICU stay and also is a risk factor for 28-day mortality, even in patients with GFR > 60 mL/min.

## 1. Introduction

Acute kidney injury (AKI) is a frequently seen clinical disorder in intensive care units (ICU). Epidemiologic studies have shown that AKI is associated with length of ICU stay, prolonged mechanical ventilation, and mortality [1-3]. Besides increasing mortality, patients who survive an episode of AKI are at high risk of progression to chronic kidney disease (CKD) [4]. Regardless of etiology, AKI is a serious medical condition affecting more than 10 million people around the world and incidence of AKI is expected to double over the next decade [5]. The incidence in the ICU population is between 20% and 30% depending on the definition used [6]. CKD, septic shock, advanced age, nephrotoxic medications such as vancomycin and colistin, critical illness, circulatory shock, burns, and surgery are well known risk factors for development of AKI in ICU [7]. The incidence of ICU-acquired AKI is higher than community-acquired AKI and the frequency of ICU-acquired AKI is also increasing over the years [8, 9]. 

AKI has been the focus of extensive basic and clinical research over the last decades. Any novel factor for accurately predicting AKI would be beneficial for preventing morbidity and mortality. Biomarkers such as neutrophil gelatinase associated lipocalin (NGAL) [10, 11] and cell cycle arrest markers tissue inhibitor of metalloproteinase-2 (TIMP-2) and insulin-like growth factor binding protein 7 (IGFBP-7) [12] have been studied for AKI stratification and prediction. However, all these biomarkers are expensive and their clinical values still appear to be limited [13].

Proteinuria is a well-known risk factor for CKD progression, cardiovascular disease and all-cause mortality in general population [14] but its effect on incidence of AKI and mortality in ICU patients is not well defined. Proteinuria can be easily and rapidly screened by a dipstick test. Studies were designed to demonstrate the relationship between proteinuria and AKI in recent years, but some of these focused only on the association between proteinuria and AKI but not mortality, or well-known risk factors such as CKD were not excluded in these studies. Shao et al. demonstrated that hypoalbuminemia in critically ill patients is independently associated with an increased risk of development of AKI and AKI progressing to CKD [15]. Li et al. showed that proteinuria was independently associated with all stages of postcardiotomy AKI in cardiac surgery patients [16]. Han et al. demonstrated that proteinuria is associated with AKI and long-term mortality in critically ill patients [17]. 

The aim of this study was to evaluate the relationship between mild to moderate proteinuria and incidence of AKI and 28-day mortality in patients with glomerular filtration rate (GFR) less than and more than 60 mL/min.

## 2. Material and methods

### 2.1. Study population 

**Figure 1:**
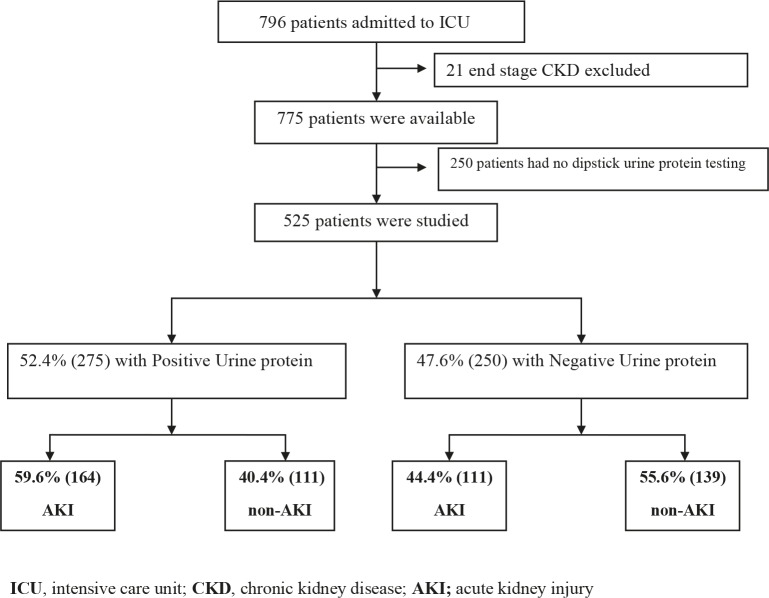
Study diagram.

This observational retrospective study was conducted in the internal medicine ICU at Hacettepe University, Ankara, Turkey, from January 2008 to April 2014. Patients who were >18 years old and had urine dipstick protein measurement within the preceding three months of ICU admission were included in the study. Exclusion criteria were severe (nephrotic range) proteinuria and end-stage kidney disease (admission GFR <15 mL/min estimated by MDRD formula) [18]. A total of 796 patients were screened. Twenty-one of 796 patients with end-stage kidney disease and 250 patients without dipstick urine protein analysis were excluded. Consequently, 525 patients were available for this analysis (Figure 1). The study was approved by the local ethics committee of Hacettepe University and was conducted in accordance with the Declaration of Helsinki. 

### 2.2. Data collection 

Hospital electronic medical records system was used for baseline information such as sex, age, comorbidities, contrast agent exposure, and calculation of Charlson comorbidity index. The 28-day mortality rates and duration of hospitalization in the ICU were determined for all patients. Data regarding creatinine, albumin, blood urea nitrogen (BUN), and urine dipstick protein within preceding three months were collected (72% of the samples were collected before hospital admission and 28% of the samples were collected at the first hospital admission) Patients’ creatinine and albumin levels were recorded at the time of ICU admission, at 48 h and 7 days after ICU admission. Urine dipstick test was used to determine proteinuria level from negative to +3 using an automated urine chemistry analyzer iChem Velocity (Beckman Coulter, Brea, CA, USA). Plasma levels of albumin and creatinine were estimated by spectrophotometry and Jaffe technique using a Beckman Coulter spectrophotometer.

AKI was defined according to the 2012 Kidney Disease Improving Global Outcomes (KDIGO) Clinical Practice Guideline for Acute Kidney Injury [19] as follows: increase in serum creatinine by ≥0.3 mg/dL within 48 h or increase in serum creatinine to ≥1.5 times baseline as stage 1 AKI; increase in serum creatinine 2–2.9 times baseline as stage 2; increase in serum creatinine more than 3 times baseline or increase in serum creatinine by ≥4 mg/dL within 48 h or initiation of renal replacement treatment.

We used the abbreviated Modification of Diet in Renal Disease (MDRD) equation to estimate the GFR [18, 20].Patients who had been admitted to ICU and had diagnosis of CKD were staged according to their MDRD estimated GFR as follows: at least 60 mL per minute per 1.73 m2, 45 to 59 mL per minute per 1.73 m2 (stage 3a), 30 to 44 mL per minute per 1.73 m2 (stage 3b), 15 to 29 mL per minute per 1.73 m2 (stage 4), and less than 15 mL per minute per 1.73 m2 (stage 5) [18].

### 2.3. Statistical analysis

Asymmetrically distributed variables in the text and tables were shown as median (minimum–maximum). For data that were not normally distributed, the Mann–Whitney U test was used if only two groups were compared; the Kruskal–Wallis one way analysis of variance was used if more than two groups were being compared. Logistic regression was used to identify variables that predict AKI. Cox regression analysis was used to identify AKI groups for mortality in AKI patients. Twenty-eight–day survival analysis was performed by Cox regression analysis. P-values less than 0.05 were considered to indicate statistical significance. Analyses were performed with SPSS 20 (SPSS, IBM, Armonk, NY) software for Windows.

## 3. Results

The study included 283 (53.9%) male and 242 (46.1%) female patients with a median age of 60 (18–95) years old. Of all the patients, 275 patients had positive urine protein and 250 patients had negative dipstick urine protein. Patients were divided into two groups as per their dipstick urine protein positivity. The characteristics of the study population were shown in Table 1. Demographic characteristics were similar in both groups. Urine protein positive group had more comorbidities including hypertension, diabetes, chronic liver disease, kidney diseases and malignancies according to Charlson comorbidity score compared to dipstick urine protein negative group (P < 0.01). There were no significant differences between two groups concerning the rates of contrast agent and nephrotoxic drug exposure (P = 0.7 and P = 0.31). 

**Table 1:** Demographic and laboratory parameters.

	Total patient(n = 525)	Positive urine protein(n = 275)	Negative urine protein(n = 250)	P-valuealue
SexMale	283 (53.9%)	143(52%)	140 (56%)	0.35
Age years18–3536–65>65	60(18-95)93(17.7%)211(40.2%)221(42.1%)	63(18-95)39(14.2%)112(40.7%)124(45.1%)	59(18-93)54(21.6%)99(39.6%)97(38.8)	0.060.060.060.03
Contrast agentYes	216(41.1%)	111(40.4%)	105(42%)	0.7
Nephrotoxic agent	335(63.8%)	181(65.8%)	154(61.6%	0.31
Charlson comorbidity score(median)	3(0-10)	3(0-10)	2(0-9)	<0.01
Diabetes	129(24.6%)	76(27.6%)	53(21.2%)	0.08
Hypertension	142(27%)	85(30.9%)	57(22.8%)	0.04
CHF (NYHA 3-4)	104(19.8%)	60(21.8%)	44(17.6%)	0.22
Malignancy	125 (23.8%)	59 (21.4%)	66 (26.4%)	0.12
SurgeryCardiacNoncardiac	3(0.6%)45(8.6%)	2(0.07%)30(10.9%)	1(0.4%)15(6%)	
AKITotalICU-acquiredBefore ICU	275(52.4%)102(19.4%)173(33%)	164(59.6%)60(21.8%)104(37.8%)	111(44.4%)42(16.8%)69(27.6%)	0.010.140.01
Hemodialysis	64(12.2%)	48(17.5%)	16(6.4%)	<0.01
Chronic kidneyNoYesG3aG3bG4	455(86.7)70(13.3%)26(5%)25(4.8%)19(3.7%)	233(84.7%)42(15.3%)8(2.9%)18(6.5%)16(5.9%)	222(88.8%)28(11.2%)18(7.2%)7(2.8%)3(1.2%)	0.170.020.04<0.01
ICU admissionSepsisRespiratory failureHypervolemiaIntoxicationOthers	140(26.7%)268(51%)10(1.9%)19(3.6%)88(16.8%)	87(31.6%)134(48.7%)1(0.4%)6(2.2%)47(17.1%)	53(21.2%)134(53.6%)9(3.6%)13(5.2%)41(16.4%)	<0.010.13<0.01
LaboratoryCre-0 (mg/dL)Cr-48 (mg/dL)Alb-0 (g/dL)Alb-48 (g/dL)Hemoglobin (g/dL)	0.9(0.1-5.9)0.9(0.2-11.2)2.9(05-6.6)2.8(1.18-4.6)11.7 (9.9-15.2)	1.06(0.2-5.9)1.1(0.2-6.97)2.74(0.54-5.03)2.57(1.18-4.32)11(9.9-12.9)	0.9(0.1-4.2)0.8(0.2-11.2)3.1(1.12-4.6)3.02(1.69-4.6)11.8(10.5-15.2)	0.030.02<0.01<0.010.1
Length of hosp.	17(0-392)	19(0-186)	16(0-392)	0.43
Length of ICU	6(0-186)	7(0-186)	6(0-54)	0.1
28-day mortality	155(29.5%)	101(36.7%)	54(21.6%)	<0.01

G3a: GFR 60–45 mL/min, G3b: GFR 45–30 mL/min, G4: GFR 30–15 mL/min, Cre: Creatinine, Alb: Albumin, ICU: Intensive care unit, CHF: Congestive heart failure, AKI: Acute kidney injury

Patients with dipstick urine protein positivity on admission had higher proportion of AKI and hemodialysis compared to dipstick urine protein negative group [164 (59.6%) vs. 111 (44.4%) and 48 (17.5%) vs. 16 (6.4%), P = 0.01 and P < 0.01, respectively] (Table 1). Median albumin level at ICU admission was significantly lower in urine dipstick protein positive group than urine dipstick protein negative group (2.74 mg/dL (0.54–5.03) vs. 3.1 mg/dL (1.12–4.6) and P < 0.01). CKD rate was similar between two groups [42 (15.3%) vs. 28 (11.2%), P = 017). Median length of ICU and hospital stay were similar between two groups (P = 0.1 and P = 0.43). Twenty-eight–day mortality rate was significantly higher in the dipstick-urine-protein-positive group compared to the negative group [101 (36.7%) vs. 54 (21.6%), P < 0.01]. A separate analysis was carried out in patients with GFR > 60 mL/min (Table 2). Similar to the total study population, Charlson comorbidity score, hypertension, rates of AKI, hemodialysis, and 28-day mortality were statistically higher in the urine-protein-positive group (P < 0.001, P = 0.01, P = 0.02, P = 0.02 and P < 0.001, respectively) even in patients with GFR > 60 mL/min.

**Table 2:** Subgroup analysis of GFR > 60 patients.

	Total patient(n = 455)	Positive urine protein(n = 233)	Negative urine protein(n = 222)	P- value
Sex Male	248(54.5%)	125(53.6%)	123(55.4%)	0.77
Contrast agent	193(42.4%)	96(41.2%)	97(43.7%)	0.63
Toxic agent	285(62.6%)	150(64.3%)	135(60.8%)	0.44
AKI	217(47.6%)	124(53.2%)	93(41.8%)	0.02
Hemodialysis	44(9.6%)	30(12.8%)	14(6.3%)	0.02
28-day mortality	133(29.3%)	89(38.2%)	44(19.8%)	<0.001

Logistic regression was used to analyze independent risk factors for AKI (Table 3). Nephrotoxic medication use (odds ratio (OR): 2.216, 95% confidence interval (CI:): 1.475–3.329), dipstick urine protein positivity (OR: 1.559, 95% CI: 1.0106–2.313) and presence of chronic kidney disease (OR: 5.883, 95% CI: 3.012–11.490) were significantly associated with AKI. However, sex (OR: 0.954, 95% CI: 0.419–1.389), diabetes (OR: 1.061, 95% CI: 0.676–1.666), hypertension (OR: 0.838, 95% CI: 0.493–1.427), and congestive heart failure (OR: 1.135, 95% CI: 0.637–2.025) were not associated with AKI. Contrast agent use appeared as a protective factor for AKI development [OR: 0.625, 95% CI: 0.419–0.993]. Similar risk factors such as nephrotoxic medication use (OR: 2.214, 95% CI: 1445–3.393) and dipstick urine protein positivity (OR: 1.493, 95% CI: 1.014–2.199) were also significantly associated with AKI in patients with GFR > 60 mL/min.

**Table 3:** Logistic regression for independent risk factors for AKI.

	Total patients (n: 525)		GFR > 60 mL/min patients ( n: 455)	
Variable	Odds ratio (95%Cl)	P-value	Odds ratio (95%Cl)	P-value
Male	0.954(0.655-1.389)	0.8	1.165(0.789-1.720)	0.44
Contrast agent	0.625(0.419-0.933)	0.02	0.538(0.354-0.818)	0.004
Nephrotoxic medication	2.216(1.475-3.329)	<0.01	2.214(1.445-3.393)	<0.01
Urine protein	1.559(1.106-2.313)	0.01	1.493(1.014-2.199)	0.04
Chronic kidney (GFR < 60 mL/min)	5.883(3.012-11.490)	<0.01		
Diabetes	1.061(0676-1.666)	0.73	0.994(0.617-1.601)	0.98
Hypertension	0.838(0.493-1.427)	0.5	1.330(0.838-2.112)	0.22
Surgery	0.903(0.504-1.616)	0.73	0.936(0.491-1.784)	0.84
Congestive heart failure	1.135(0.637-2.025)	0.66	1.524(0.885-2.625)	0.12

**Figure 2:**
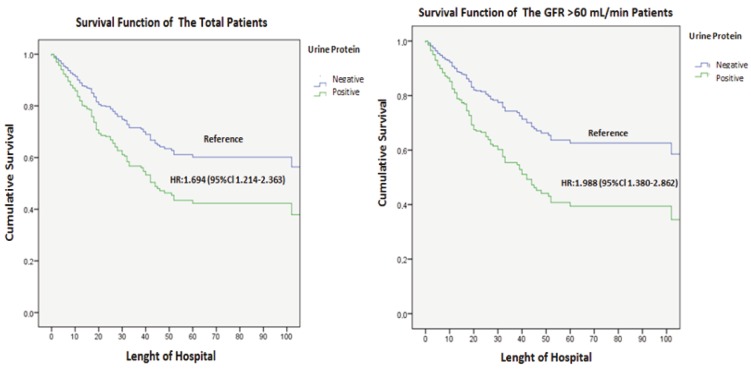
Cox regression analysis: 28-day hospital mortality according to the urine protein positivity for the whole group (P = 0.002) and with GFR > 60 mL/min (P < 0.001).

Urine dipstick protein positivity was a significant predictor of 28-day hospital mortality in univariate cox regression analysis. Compared with negative urine dipstick test, the adjusted hazard ratio (HR) of proteinuria was 1.694 (95% CI: 1.214–2.363). After exclusion of the patients with GFR <60 mL/min, urine dipstick protein positivity was still a significant predictor of mortality as compared with negative urine dipstick protein (HR: 1.988 (95% CI: 1.380–2.862) (Figure 2).

## 4. Discussion

AKI is a common disorder in critically ill patients and has been associated with increased mortality and length of ICU stay [1, 21]. Various epidemiologic studies showed the incidence of AKI was about 3%–18% [22]. Most of patients in ICUs are exposed to more than one risk factor such as contrast agent, CKD, and nephrotoxic medications for AKI. This study demonstrated that dipstick urine protein positivity is related to increased risk of AKI and 28-day mortality in ICU patients. Results also support previous epidemiologic studies that nephrotoxic medication and CKD were independent risk factors for AKI. On the contrary, contrast agent exposure seems to have not a detrimental, but a protective effect against AKI development. This might be explained either by introducing preventive precautions (such as IV hydration or N-acetylcysteine administration) before contrast exposure or the observational and retrospective nature of this trial because preventive precautions are taken to reduce the development of contrast nephropathy in accordance with international standards for all patients in ICU where the data were collected. Moreover, some recent studies have shown no increased risk of AKI due to contrast exposure in low-risk patients [23].

Regardless of etiology, AKI is a serious medical condition affecting more than 10 million people around the world [5] and associated with length of ICU stay, prolonged mechanical ventilation and mortality [1-3]. Any novel factor for accurately predicting AKI would be advantageous. Over the past decade many studies have focused on the independent risk factors [24-26] and early predictors for AKI [27, 28]. Novel potential biomarkers such as cystatin C [29], interleukin-18 (IL-18) [27], and NGAL, [30] have failed to demonstrate clear clinical utility [31]. These biomarkers are not only difficult to measure, but also very expensive for routine clinical use. In this study, simple assessment of dipstick protein was found to be a predictor of developing AKI. In a previous study, Han et al. demonstrated that proteinuria is associated with AKI and long-term mortality in critically ill patients [17]. As CKD has been shown to be significant risk factor for developing AKI in previous studies [2, 32, 33], Han et al. did not exclude patients with GFR < 60 mL/min. Unlike Han et al.’s, in this study, AKI risk factors in patients with GFR > 60mL/min were separately analyzed and it was found that urine protein positivity was still a significant risk factor for AKI.

This study showed that dipstick urine protein positivity was associated with not only developing AKI, but also increased 28-day mortality in critically ill patients. There is limited data about association between proteinuria and mortality in the literature. Hara et al. reported that proteinuria was a simple sign of coexisting systemic inflammation and it was associated with mortality in patients with non-Hodgkin lymphomas [34]. Konta et al. showed that proteinuria/albuminuria were independently associated with mortality in the Japanese general population [35]. Ota et al. and Tonelli et al. also investigated all-cause mortality in acute myocardial infarction (AMI) patients [36, 37]. Both studies showed that dipstick proteinuria was an independent predictor of all-cause mortality with or without impaired kidney function. The mechanisms responsible for the association between proteinuria in critically ill patients and mortality remain unclear. However, inflammation and endothelial dysfunction are proposed mechanisms for this relationship [38]. Studies using the inflammatory cytokines interleukin-6 (IL-6) C-reactive protein, and tumor-necrosing-factor alfa (TNF-α) have shown that regardless of the presence of diabetes these markers are associated with the occurrence of microalbuminuria and mortality [39, 40]. 

This study has some limitations. First, we cannot generalize our findings outside critically ill patients. Second, we have no detailed information on some interventions and medical conditions (e.g., dosage of diuretic usage and urinary tract infection) that could induce AKI and false urine protein positivity. This limitation could be overcome by more than one proteinuria measurement in future studies. Third, assessment for AKI stopped at day 7 in our study, and it is possible that we missed events of AKI occurring later. Fourth, we could not evaluate the effect of ICU on proteinuria as the patients with proteinuria outside the ICU were not included in the study. Finally, hemodynamic data of patients were not recorded. However, management of patients in ICU is carried out in accordance with international guidelines. Therefore, both groups could be considered to be similar in terms of hemodynamic stability. Furthermore, studies addressing these limitations with larger sample size and involvement of multicentric trials are needed to evaluate the crucial effect of proteinuria on development of AKI and mortality.

In conclusion, proteinuria before ICU admission is a risk factor for development of AKI within seven days of ICU stay and also is a risk factor for 28-day mortality, even in patients with GFR > 60 mL/min.
